# Thrush

**Published:** 1904-12

**Authors:** 


### THRUSH.

This disease, which is so common among children, is generally due to uncleanliness on the part of the nurse or parent, the *oidium albicans* being readily introduced and rapidly propagated in children's mouth (Illoway *Medical News*, No. 17, *Journal American Medical Association*, March 12, 1904). The treatment of the milder forms is easy with hyposulphite or borax, but in the severer forms Illoway has had the best results with a mixture of tincture of iodine and glycerine, half dram of the former to three and a half of the latter, applied locally to the parts with a camel's hair pencil. Two cases are reported. He thinks the glycerine is an important factor in the treatment. It loosens up the mycelium and makes its removal sure. This plan, he thinks, surpasses any that has heretofore been recommended in its readiness of application, and in its harmlessness and rapidity of action.

